# Eighteenth Annual Convention T. S. M. A.

**Published:** 1886-03

**Authors:** 


### EIGHTEENTH ANNUAL CONVENTION T. S. M. A.

FOR years, the Texas State Medical Association had a mere nominal existence, and hung together, with a small membership, only through a kind of enforced enthusiasm on the part of a few zealous workers. With the extension of railroads, and the rapid settling up of the country, and latterly the successful inauguration of a live Home Medical Organ, it took on a newness of life, and its growth and progress in the last four or five years have only been exceeded by the awakening of interest in medical matters everywhere witnessed and experienced. The T. S M. A. may be said to be on a boom! Its published volumes of Transactions have been applauded by the medical profession of America and by the medical press, not alone for the excellence of its get up, mechanically, but for the excellence, and number, and variety of the papers written and contributed by its live members. The Transactions for 1885 have been pronounced by some of our exchanges as second only to that of the New York State Medical Association; while its papers have been copied into the medical journals far and near.

This year there will be a larger and more enthusiastic attendance than usual. We learn from our canvassers throughout the State, that there is a general and wide spread interest being manifested in the approaching meeting, and that "everybody is going." Well, they should go. There are more inducements to go than usual. First, Dallas is a central railroad point; can be reached from any point, and, no doubt, reduced rates will be secured; it is centrally located; it is a fine and attractive city, noted for its hospitality. The committee of arrangements have spread themselves, and such an entertainment as will be given the delegates, was never before seen; and the ladies, too, will grace the occasion with the charm of their presence; 2nd, it is understood that all delinquent dues for which members have been suspended, will be remitted, and suspended members reinstated on the same basis as new members. We suggested that, as an inducement, and the President informs us he will recommend it in his message, which is tantamount to having it done; 3rd, the State Pharmaceutical Association meets at the same time and place; also the Physicians' Mutual Benefit Association of Texas will bold a meeting of members present and elect a board of directors, etc.

At this meeting of the T. S. M. A. it is supposed the organization of the 9th International Medical Congress will be discussed. This alone will attract many. Delegates are to be elected to the convention of the American Medical Association at St. Louis, which occurs only one week later, to-wit: May 4. We hope our friends will lay down the saddlebag and all else, and meet with us. Christmas comes but once a year.

